# Expression of Caspase-3 in Circulating Innate Lymphoid Cells Subtypes Is Altered by Treatment with Metformin and Fluvastatin in High-Fat Diet Fed C57BL/6 Mice

**DOI:** 10.3390/cells11091430

**Published:** 2022-04-23

**Authors:** Vuyolwethu Mxinwa, Bongani B. Nkambule, Tawanda M. Nyambuya, Phiwayinkosi V. Dludla

**Affiliations:** 1 School of Laboratory Medicine and Medical Sciences (SLMMS), College of Health Sciences, University of KwaZulu-Natal, Durban 4013, South Africa; mxinwav@ukzn.ac.za; 2 Department of Health Sciences, Namibia University of Science and Technology, Windhoek 9000, Namibia; mnyambuya@nust.na; 3 Biomedical Research and Innovation Platform, South African Medical Research Council, Tygerberg 7505, South Africa

**Keywords:** obesity, prediabetes, type 2 diabetes, circulating innate lymphoid cells, haematology, metabolism, metformin, fluvastatin

## Abstract

The current study aimed to determine the expression levels of caspase-3 in circulating innate lymphoid cell subtypes (ILCs) in a high-fat diet (HFD)-induced prediabetes mouse model. Another critical point was to assess the therapeutic effects of metformin and fluvastatin in modulating caspase-3 activation in ILCs within these HFD-fed mice. Prominent results showed that mice exposed to HFD for 14 weeks displayed impaired glucose tolerance that was accompanied by elevated levels of low-density lipoprotein cholesterol (LDL-c) and altered haematological profile as characterised by significantly increased concentrations of red blood cell count, white cell count and lymphocytes when compared to those fed a low-fat diet (LFD). Moreover, the expression of caspase-3 in ILC1 and ILC3 was significantly increased in the HFD groups in comparison to the LFD-fed group. Notably, six-week treatment with metformin and fluvastatin reduced the caspase-3 activation in ILC subtypes. The reduced caspase-3 activation in ILC1 was inversely associated with HDL-c levels following metformin treatment. Interestingly, the reduced caspase-3 activation in ILC3 was associated with lower total cholesterol following fluvastatin treatment in these HFD-fed mice. However, there were no differences in activation of caspase-3 on ILC2 or any association between caspase-3 activation and changes in body weight or fasting blood glucose. Thus, while HFD-feeding clearly modulates ILCs, potentially leading to pro-apoptotic mechanisms, metformin and fluvastatin may play a major role in protecting against such metabolic disturbances.

## 1. Introduction

Type 2 diabetes (T2D) contributes to the development of cardiovascular complications, which have become a major cause of morbidity and mortality worldwide [1,2]. In fact, cardiovascular disease (CVD)-related mortality is estimated to be at least two times higher in individuals with T2D in comparison to healthy adults [3]. Obesity plays a central role in the incidence T2D-associated CVD events, especially through its associated metabolic complications including impaired glucose tolerance, insulin resistance, dyslipidaemia, and a state of chronic inflammation [4,5]. Obesity is associated with excessive adiposity accompanied by the activation of pro-inflammatory immune cells such as tissue resident innate lymphoid cells (ILCs) and macrophages [6]. The activation of ILC or macrophages can potentially lead to an abnormal release of pro-inflammatory cytokines such as tumour necrosis alpha (TNF-α) and interleukin 6 (IL-6) that promote poor insulin sensitivity [6,7].

The ILCs are differentiated into three main categories, namely ILC1, ILC2 and ILC3—depending on their metabolic or immune-modulatory function [8,9]. For example, ILC1s are responsible for type 1 immune response, by mainly secreting the interferon gamma (IFN-γ) that can fend off intracellular bacteria and viruses through activation of macrophages [9,10]. Similarly, ILC2/ILC3s have emerged as a vital component of an immune response by largely contributing to the secretion of essential cytokines, which are important for provoking a pro-inflammatory response during the development of diverse diseases [8,9]. Available evidence even acknowledges that abnormalities in the frequency or the levels of activated peripheral natural killer cells, including ILCs, are consistent with the development and propagation of metabolic complications, including diabetes [11,12]. Recently, in an animal model of diet-induced obesity, ILC2s regulated chronic inflammation by modulating saturated fatty acid absorption in visceral adipose tissue [13]. Pro-apoptotic mechanisms, especially the regulation of caspase-3, within the circulating ILCs, has already been acknowledged to play a major role in the development of commonly investigated diseases, including cancer and human immunodeficiency virus (HIV) [14,15]. However, such information has been rarely explored in connection to the development of metabolic conditions such as T2D. This is in fact important to understand, since it has already been reported that metformin can exert beneficial effects by suppressing cellular apoptosis or blocking caspase-3 activation in experimental conditions of T2D [16,17]. 

Glucose- and lipid-lowering drugs such as metformin and statins improve metabolic function and alleviate T2D-related complications [18,19]. In our previous work, we explored the therapeutic benefits of using commonly used antidiabetic drugs such as metformin to combat hyperglycaemia-induced complications [20,21]. Importantly, animal models of prediabetes using obesogenic diets have become a relevant model to characterise and determine the impact of high-fat diet (HFD)-feeding on circulating ILCs in exacerbating inflammation in a state of impaired glucose tolerance [22]. In addition to the well-described role of ILCs in immune response and chronic inflammation [8,9], it remains critical to determine how ILCs modulate other metabolic processes such as apoptosis, which is involved in tissue damage [23]. Thus, besides establishing an association between HFD-feeding and the level of caspase-3 activation on ILCs, the current study provides essential evidence on the therapeutic effects of commonly used antidiabetic drugs (metformin and fluvastatin), using obesogenic diet to mimic conditions of obesity and metabolic syndrome.

## 2. Materials and Methods

### 2.1. Animals and Animal Handling

Twenty-eight male C57BL/6 mice were purchased from the Biomedical Resource Unit (BRU) at the University of KwaZulu-Natal (UKZN). The mice were housed at the BRU in a temperature-controlled 12 h light/dark cycle. The mice were allowed free access to water throughout the experimental period. The study received ethical approval from the University of KwaZulu-Natal Animal Research Ethics Committee (AREC), with the registration number AREC/081/018D.

### 2.2. Study Design and Measurement of Basic Metabolic Parameters

Briefly, in this study, C57BL/6 mice were grouped into 4 groups: the low-fat diet (LFD) group, HFD group, as well as groups first fed a HFD then metformin (150 mg/kg) or fluvastatin (20 mg/kg). The mice in the control groups were fed LFD (10% fat; Research Diets #D12450J) or HFD (60% fat; Research Diets #D12492) for a period of 14 weeks, whereas treated animals received HFD for 8 weeks and then received fluvastatin or metformin (by oral gavage) for an additional 6 weeks. The choice of HFD-induced obesity model, including relevant treatments, which included metformin and fluvastatin (treatment drugs were dissolved in water), was done following previously described methods [21,24]. Weekly animal body weights were monitored throughout the duration of the experiment. To evaluate glucose metabolism (oral tolerance tests), the animals were fasted for 8 h, and 3 g/kg glucose solution was administered through oral gavage, whereas basal glucose levels and postprandial glucose levels were measured at varying time intervals (0, 15, 30, 60, 90, 120 min) using the OneTouch^®^Select^®^ handheld glucometer (LifeScan Inc., Milpitas, CA, USA). Additionally, serum insulin levels (50 μL) were also determined following an 8 h fast at the end of the experimental phase (week 14) using an enzyme-linked immunosorbent assay (ELISA) kit (Thermo Fisher, Waltham, MA, USA) as per the manufacturer’s protocol. 

### 2.3. Measurement of Haematological Indices and Lipid Profiles

The lipid profiles, including high-density lipo-proteins (HDL) and low-density lipo-proteins (LDL), as well as total cholesterol levels (50 μL) were measured from serum using an ELISA kit (Abcam, Cambridge, MA, USA) as per the manufacturer’s protocol. The haematological indices, including red blood cell count (RBC), white blood cell count (WCC), lymphocytes and platelet count, were measured after 5 weeks of treatment using the Beckman Coulter Ac T diffTM hemo-analyzer (Beckman Coulter, Brea, CA, USA) as per the manufacturer’s protocol.

### 2.4. Measurement of Caspase-3 Expression on ILC Subtypes

**Figure 1:**
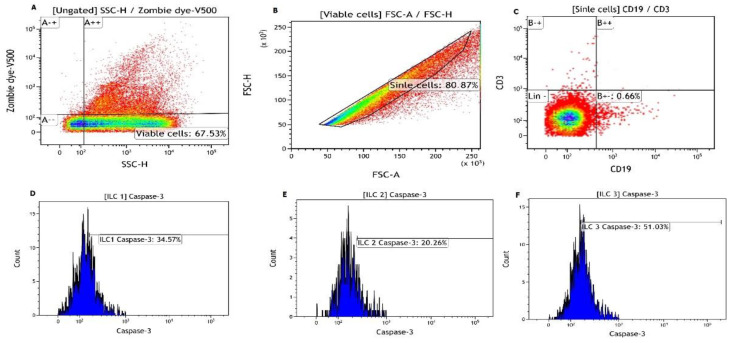
Gating strategy used for the measurement of caspase-3 expression in circulating innate lymphoid cells. To detect the active form of caspase, the kits take advantage of FLICA methodology, which is an affinity label. Briefly, the FLICA reagent is thought to interact with the enzymatic reactive centre of an activated caspase via the recognition sequence and then to attach covalently through the FMK moiety. (**A**) illustrates the viable and non-viable cells from monocyte and granulocyte depleted peripheral blood mononucleated cells (PBMCs). (**B**) shows the singlets gated from the viable cells in (**A**). (**C**) shows lineage marker negetive lymphocytes that are gated from sigletes. (**D**–**F**) illustrates caspase-3 expression on innate lymphoid cells subtypes (ILC)1, ILC2 and ILC3 gated from lineage negative viable cells.

Briefly, to measure the expression of caspase-3 (in peripheral blood mononucleated cells—PBMCs) on ILCs, monocytes and granulocytes were depleted using Anti-CD11b (Clone M1/70) and Anti-Ly-6G and Ly-6C (Clone RB6-8C5) magnetic cell particles (BD Bioscience, San Jose, CA, USA), following the lymphocyte enrichment protocol using the BD IMAG system (BD Biosciences, San Jose, CA, USA). To quantify the different ILC subtypes, isolated lineage marker negative lymphocytes were gated (CD19-CD3-), and the expression of CD55 was used to determine ILC1 (Figure 1). Whereas ILC2s were gated based on lack of lineage markers and expression of CD117, ILC3s were gated and identified based on the lack of expression of lineage classical markers (CD19, CD3, CD117, CD55) and expressions of CD4 (Figure 1). Vibrant FAM Caspase-3 and 7 Assay kit (Thermo Fisher, Waltham, MA, USA) was used to quantify the level of expression and activity of caspase-3. Please note that to detect the active form of caspase, the kits take advantage of FLICA methodology, which is an affinity label. Briefly, the FLICA reagent is thought to interact with the enzymatic reactive centre of an activated caspase via the recognition sequence and then to attach covalently through the FMK moiety. All the data were acquired using BD FACS Aria III flow cytometer and analysed using FlowJo version 10.6.2 analysis software (BD Biosciences, San Jose, CA, USA).

### 2.5. Statistical Analysis

All statistical analysis was performed using GraphPad Prism 8 (GraphPad Software Inc., San Diego, CA, USA). A two-way analysis of variance (ANOVA) was used for parametric comparison of data across the groups, followed by the Tukey test. Parametric data were presented as mean and standard deviation (SD). The Kruskal-Wallis test followed by the Dunn’s multiple comparison test were applied in non-parametric data and the results were presented as median and interquartile range (IQR). The correlation analysis between the caspase-3 expression and the corresponding metabolic parameters was performed using the Pearson and Spearman coefficients. A *p*-value of ˂ 0.05 was considered statistically significant.

## 3. Results

### 3.1. Impact of HFD-Feeding on Basic Metabolic Parameters

We first assessed the impact of HFD on basic metabolic parameters, such as body weights, fasting plasma glucose levels and lipid profiles. Briefly, a post hoc analysis showed higher body weight in the HFD (29.71 ± 0.29), *p* < 0.001 compared to the LFD group (25.42 ± 0.48) (Table 1). Similarly, we showed that fasting blood glucose levels were statistically different across the experimental groups (F _(3,18)_ = 7.94, *p* < 0.001) (Table 1). A post hoc test showed higher fasting glucose levels in HFD (6.37 ± 0.75), *p* < 0.001 compared to the LFD group (4.40 ± 0.39) (Table 1). Consistently, the area under the curve (AUC) was significantly different across experimental groups (F _(2,18)_ = 8.52, *p* < 0.001) (Table 1). A post hoc test showed higher fasting glucose levels in HFD (959.4 ± 75.34), *p* < 0.01 compared to the LFD group (826.9 ± 79.53) (Table 1).

### 3.2. Impact of Treatment with Metformin and Fluvastatin on Basic Metabolic Parameters

Next, we determined the impact of treatment with metformin and fluvastatin on basic metabolic parameters. Data showed that short-term treatments significantly reduced animal body weight in the metformin (22.00 ± 0.53), *p* < 0.001 and fluvastatin (21.71 ± 0.47) *p* < 0.001 groups in comparison to the HFD group (29.71± 0.29) (Table 1). Furthermore, the post hoc test showed that metformin (4.07 ± 1.18), *p* = 0.0015 lowered the fasting blood glucose levels compared to the HFD group (Table 1). However, the fasting glucose levels in the fluvastatin and HFD-fed group were not markedly different (Table 1). Additionally, we showed a significant reduction in the AUC following treatment with metformin (803.8 ± 25.81), *p* < 0.001 and fluvastatin (855.9 ± 55.39), *p* < 0.01 in comparison to the HFD group (959.4 ± 28.47) (Table 1).

### 3.3. Impact of HFD and Treatment with Metformin and Fluvastatin on Lipid Profiles

Notably, lipid profiles, including determining the levels of total cholesterol, LDL-c and HDL-c remains essential to determine the risk of CVD in conditions of metabolic syndrome. First, it was clear that there were significant differences in the levels of total cholesterol (F _(3,15)_ = 13.89, *p* < 0.001), LDL-c (F _(3,15)_ = 30.57, *p* < 0.001) and HDL-c (F _(3,15)_ = 3.93, *p* < 0.01) across the groups (Table 1). The levels of total cholesterol and HDL-c were partially increased in the HFD compared to the LFD group. Additionally, the LDL-c levels were significantly increased in the HFD group compared to the LFD group (*p* < 0.001). Then, a post hoc test showed that metformin (*p* < 0.001) and fluvastatin (*p* < 0.001) significantly lowered the levels of total cholesterol compared HFD group (Table 1). Additionally, a post hoc test showed that metformin (0.05 ± 0.01), *p* < 0.001 and fluvastatin (0.06 ± 0.02), *p* < 0.001 treatments markedly reduced the levels of LDL-c compared to HFD (0.12 ± 0.01) (Table 1). Lastly, metformin (0.04 ± 0.01), *p* < 0.05 had reduced HDL-c levels compare to the HFD group (0.06 ± 0.01), whilst the fluvastatin (0.06 ± 0.01) treatment group did not significantly affect HDL-c (Table 1).

### 3.4. Impact of HFD and Treatment with Metformin and Fluvastatin on Basic Haematological Indices

Generally, determining the levels of basic haematological indices such as WCC, lymphocytes and RBC is important to assess in conditions of metabolic syndrome. Here, data showed there were significant differences in the levels of circulating WCC (F _(3,15)_ = 56.69), *p* < 0.001 across the groups (Table 1). A post hoc test showed that HFD group (7.89 ± 0.89), *p* < 0.001 had higher levels of WCC compared to the LFD (2.06 ± 0.32), whilst the metformin (6.71 ± 2.54), *p* < 0.05 and fluvastatin (5.82 ± 1.38), *p* < 0.01 had reduced these levels compared to HFD (7.89 ± 0.89) (Table 1). Furthermore, the two-way ANOVA test showed that there were significant changes in lymphocyte count (F _(3,18)_ = 5.75, *p* < 0.01) across the treatment groups (Table 1). A post hoc analysis showed higher lymphocyte count in HFD (88.57 ± 2.03), *p* < 0.05 compared to the LFD group (82.51 ± 1.74), whilst the metformin and fluvastatin did not reduce the lymphocyte count (Table 1). Lastly, there were significant changes in the RBCs (F _(3,15)_ = 16.36). A post hoc test showed that the HFD group (8.47 ± 0.92), *p* < 0.001 had higher levels of RBCs compared to the LFD group (5.63 ± 0.83), whilst metformin (7.09 ± 0.86), *p* < 0.05 and fluvastatin (6.52 ± 0.97), *p* < 0.001 reduced the levels of RBCs.

### 3.5. Impact of HFD and Treatment with Metformin and Fluvastatin on the Expression of Caspase-3 on ILCs

Next, we evaluated the effect of HFD as well as treatments with metformin and fluvastatin on the expression of caspase-3 in circulation ILC subtypes. The results showed there were significant differences in the levels of caspase-3 expression on circulating ILC1 (F _(3,15)_ = 7.57), *p* = 0.0026 across the groups (Table 2). Notably, there was an increase in expression of caspase-3 in the HFD (47.06 ± 11.08) compared the LFD group (20.61 ± 6.59), *p* = 0.0028 (Table 2). Similarly, the metformin (43.50 ± 12.19) treatment increased the expression of caspase-3 on circulating ILC1 compared to the LFD untreated (20.61 ± 6.59), *p* = 0.0089, but it was not significant compared to the HFD group (Table 2). The fluvastatin treatment did not alter the expression of caspase-3 on circulating ILC1. There were significant differences in the levels of caspase-3 expression on circulating ILC2 (F _(3,15)_ = 5.55), *p* < 0.01 across the groups. The post hoc test showed the HFD feeding did not alter the expression of caspase-3 on circulating ILC2 (Table 2). However, the fluvastatin (28.84 ± 11.01) significantly increased the caspase-3 on circulating ILC3 compared to the LFD untreated (5.69 ± 4.37), *p* < 0.01 but no significant difference was observed compared to the HFD group (Table 2). Lastly, there were significant differences in the levels of caspase-3 expression on circulating ILC2 (F _(3,15)_ = 7.23), *p* < 0.01 across the groups. The HFD feeding (32.05 ± 5.02) significantly increased the expression of caspase-3 on circulating ILC3 when compared to LFD untreated (21.98 ± 4.37), *p* < 0.05 (Table 2). The metformin (35.59 ± 6.35) also increased the expression of caspase-3 on ILC3 compared to the LFD group (21.98 ± 4.37), *p* = 0.0063, but not compared to HFD group. The fluvastatin (23.64 ± 5.22) also reduced the expression of caspase-3 on ILC3 compared to the HFD group (32.05 ± 5.02), *p* < 0.05.

### 3.6. Impact of HFD and Treatment with Metformin and Fluvastatin on the Expression of Caspase-3 on ILCs

To assess associations between caspase-3 expression and metabolic parameters, we explored the relationship between caspase-3 levels on ILCs and body weight, fasting blood glucose and total cholesterol levels (Table 3). In the metformin group, HDL-c levels were inversely associated with caspase-3 levels on circulating ILC1s (r = −0.82, *p* < 0.05). Similarly, TC levels were also strongly associated with decreased levels of caspase-3 expression on circulating ILC3s (r = −0.93, *p* < 0.001) (Table 3).

## 4. Discussion

Metformin and fluvastatin represent some of the commonly used therapies to alleviate T2D-related complications [25,26], especially the state of hyperglycaemia and dyslipidaemia. Although these therapies have been used for decades, their mode of action remains to be fully evaluated. This is especially true in relation to understanding their effects in modulating metabolic functions of ILCs [13]. This work further extends from our recent report indicating that characteristic features of T2D such as impaired glucose tolerance and elevated total cholesterol levels occur consistently with increased levels of circulating ILC1s in mice fed HFD for 8–13 weeks [22]. This result generally highlights the broader function of ILCs as potential therapeutic targets for improving metabolic function, separate from their established role in inflammatory and autoimmune disorders [27]. 

In fact, due to their emerging role in metabolism [13], the first aim of the current report was to establish the impact of HFD-feeding on caspase-3 activation on circulating ILCs. The major results showed that HFD-feeding promoted caspase-3 activation within circulating ILCs1 and ILCs3 of mice that were fed a HFD in comparison to those on LFD. Interestingly, this consequence was consistent with impaired glucose tolerance, elevated levels of LDL-c and altered haematological profile, as characterised by significantly increased concentrations of RBC, WCC and lymphocytes in these HFD-fed mice. Further inciting this, besides the accomplished pathological influence of elevated haematological indices on low-grade inflammation [22,28], ILCs may be an important feature that is implicated in the regulation of distinguished metabolic processes, including programmed cell death or apoptosis. Although necessary for cellular life forms, apoptosis may be exacerbated in a state of T2D, leading to various complications such as pancreatic β-cell dysfunction and CVDs [29,30]. 

Caspases are cysteine-aspartyl specific proteases, which are known to play a key role during the activation of apoptosis [31] in addition to their involvement in aggravated pro-inflammatory response [32]. Although actively studied for their role in regulating apoptosis in cancer or during tumour development [15,33], very few studies have reported on the activation of caspases on circulating ILCs within a state of metabolic disease. Thus, the significance and broader function of ILCs during the pathogenesis of metabolic diseases such as T2D is highlighted, as reflected by our mouse model of HFD-induced glucose intolerance. Importantly, the current findings lay an important foundation for future research to looking at the correlation between tissue-specific expression of caspase-3 and the frequency of ILCs during the development of T2D, especially since there were no differences in terms of activation of caspase-3 in ILCs2 after HFD feeding. Nonetheless, the current results indicate that impaired glucose metabolism is consistent with activation of cell death pathways through multiple mechanisms that may involve activation of caspase-3 within ILCs.

Another critical point addressed by the current study is to establish the therapeutic effects of metformin and fluvastatin in modulating caspase-3 activation on ILCs within a mouse model of HFD. Besides their established properties in improving some basic metabolic parameters and lipid profiles (as demonstrated in Table 1), the results indicated that both metformin and fluvastatin increased caspase-3 activation levels on ILCs1 when compared to LFD; however, they did not significantly affect animals exposed to HFD, nor those that were untreated. A similar trend of results was observed with caspase-3 activation of on circulating ILCs2; however, metformin showed a significant effect in reducing caspsase-3 activation on circulating ILCs3 in animals fed HFD. Interestingly, fluvastatin reduced caspase-3 activation on ILC3 (as demonstrated in Table 2), which could potentially mean that the statin drug may present with anti-apoptotic properties. The potential effectiveness of fluvastatin to reduce apoptosis was further correlated with reduced cholesterol levels (as demonstrated in Table 3). This then highlights the potential therapeutic effects of fluvastatin to hinder pro-apoptotic mechanisms beyond its capacity to improve lipid metabolism, as previously reported [34]. Lastly, in mice given metformin, HDL-c levels were inversely associated with caspase-3 levels on circulating ILC1s, further indicating the importance of improving basic metabolic parameters such as HDL-c to suppress cell death, as previously reported [35,36].

## 5. Conclusions

In conclusion, HFD feeding was essential in establishing an experimental model of glucose intolerance that was used to study the frequencies of ILC subtypes, caspase-3 activation and the potential effectiveness of metformin and fluvastatin to modulate caspase-3 in ILCs. The results demonstrated that HFD feeding certainly increased ILC1 caspase-3 activation, while metformin, a glucose-lowering drug, partially reduced ILC1 caspase-3 activation. Moreover, HFD significantly increased caspase-3 activation on ILC3, and this effect was partially reduced by fluvastatin. Certainly, these results still need to be confirmed in established experimental models of T2D, especially assessing major tissues such as the heart, liver, spleen and kidney. However, these results lay an important foundation on the potential therapeutic mechanisms associated with the use of fluvastatin and metformin to modulate ILCs to improve metabolism and other related parameters.

## Figures and Tables

**Table 1:** Metabolic changes and glucose tolerance following treatments with metformin and fluvastatin.

	LFD (*n* = 7)	HFD (*n* = 7)	MET (*n* = 7)	FLUV (*n* = 7)	*p*-Value
Changes in BW (g)	25.42 ± 0.48	29.71 ± 0.29 ^aaa^	22.00 ± 0.53 ^bbb^	21.71 ± 0.47 ^bbb^	**0.001**
**Metabolic profile**					
FPG (mmol/L)	4.40 ± 0.39	6.37 ± 0.75 ^aa^	4.07 ± 1.18 ^bb^	5.20 ± 01.34	**0.01**
AUC (mmol/L×120 min)	826.9 ± 79.53	959.4 ± 75.34 ^aa^	803.8 ± 68.29 ^bb^	810.9 ± 85.41 ^bb^	**0.001**
Insulin (µU/L)	10.75 ± 2.67	10.42 ± 1.12	10.06 ± 1.00	12.19 ± 5.89	0.54
**Lipid profiles**					
TC (µg/µL)	0.08 ± 0.02	0.12 ± 0.02	0.03 ± 0.01 ^bb^	0.03 ± 0.003 ^bb^	**0.001**
LDL-c (µg/µL)	0.05 ± 0.01	0.12 ± 0.01 ^aaa^	0.05 ± 0.01 ^bbb^	0.06 ± 0.02 ^bbb^	**0.001**
HDL-c (µg/µL)	0.04 ± 0.01	0.06 ± 0.01	0.04 ± 0.01	0.06 ± 0.01	0.0298
**Haematological parameters**					
RBC (10^6^/µL)	5.63 ± 0.83	8.47 ± 0.92 ^aaa^	7.09 ± 0.86 ^b^	6.52 ± 0.97 ^bb^	**0.001**
WCC (10^3^/µL)	2.06 ± 0.32	7.89 ± 0.89 ^a^	6.43 ± 0.46 ^b^	5.82 ± 1.38 ^bb^	**0.001**
Lymphocyte	82.51 ± 1.74	88.57 ± 2.03 ^a^	84.97 ± 5.4	90.41 ± 0.83 ^aa^	**0.01**
Platelet counts (10^3^/µL)	516 ± 504.90	793.1 ± 207.5	1022.0 ± 437.0	574.3 ± 369.7	0.1086

Results expressed as mean ± SD statistical significance shown in bold. Statistical analysis is designated by ^a^ *p* < 0.05, ^aa^ *p* < 0.01, ^aaa^ *p* < 0.001 vs. LFD; ^b^ *p* < 0.05, ^bb^ *p* < 0.01, ^bbb^ *p* < 0.001 vs. HFD; MET. Abbreviations: AUC: area under the curve; BW: body weight; FLUV: fluvastatin; FPG: fasting plasma glucose; HDL-c: high density lipoproteins; HFD: high-fat diet; LDL-c: low-density lipoproteins; LFD: low-fat diet; MET: metformin; RBC: red blood cell count; TC: total cholesterol; WCC: white cell count.

**Table 2:** Caspase-3 expression on innate lymphoid cell subtypes after treatments with metformin and fluvastatin.

	LFD	HFD	MET	FLUV	*p*-Value
%ILC1 caspase-3	21 (6.59)	47 (11.08) ^aa^	44 (12.19) ^aa^	34 (9.17)	**0.01**
%ILC2 caspase-3	6 (4.37)	18 (5.42)	21 (12.79)	29 (11.01) ^aa^	**0.01**
%ILC3 caspase-3	22 (4.37)	32 (5.02) ^a^	36 (6.35) ^aa^	24 (5.22) ^c^	**0.01**

Results expressed as mean ± SD; statistical (given in parenthesis) significance shown in bold. Statistical analysis is designated by ^a^ *p* < 0.05, ^aa^ *p* < 0.01 vs. LFD; ^c^ *p* < 0.05 vs. MET. Abbreviations: LFD: low-fat diet; HFD: high-fat diet; MET: metformin; FLUV: fluvastatin. Please note that to detect the active form of caspase, the kits take advantage of FLICA methodology, which is an affinity label. Briefly, the FLICA reagent is thought to interact with the enzymatic reactive centre of an activated caspase via the recognition sequence and then to attach covalently through the FMK moiety.

**Table 3:** Associations between caspase-3 expression on innate lymphoid cells and metabolic parameters.

HFD (*n* = 7)			MET (*n* = 7)		FLUV (*n* = 7)	
	r	*p*-Value	r	*p*-Value	r	*p*-Value
ILC1						
Weight	0.39	0.4667	−0.36	0.4838	0.22	0.6781
Glucose	−0.63	0.1824	0.38	0.4595	−0.02	0.9720
HDL-c	−0.56	0.2453	−0.82	**0.05**	0.07	0.8980
LDL	−0.56	0.2453	0.34	0.5074	0.71	0.139
TC	−0.10	0.8578	−0.49	0.3239	−0.48	0.372
ILC2						
Weight	0.63	0.1764	−0.52	0.2873	0.56	0.2490
Glucose	−0.53	0.2804	0.71	0.1150	0.44	0.3863
HDL-c	−0.42	0.4106	0.08	0.8774	0.62	0.1873
LDL	0.66	0.1508	0.62	0.1881	0.52	0.2925
TC	−0.15	0.8167	−0.37	0.4747	0.15	0.7758
ILC3						
Weight	0.75	0.0886	−0.77	0.0707	−0.20	0.7064
Glucose	−0.11	0.8307	−0.09	0.8644	−0.70	0.1207
HDL-c	−0.20	0.7047	0.70	0.1183	0.14	0.7915
LDL	0.27	0.6107	0.53	0.2847	0.13	0.8032
TC	0.20	0.7004	−0.39	0.4455	−0.97	**0.01**

Statistical significance shown in bold. Abbreviations: HFD: high-fat diet; MET: metformin; FLUV: fluvastatin; LDL-c low density lipoproteins; HDL-c: high density lipoproteins; TC: total cholesterol. Boldface indicates significance.

## Data Availability

Data is contained within the article. However, raw data presented in this study are available on request from the corresponding authors.
